# Direct Detection of FoxP3 Expression in Thymic Double-Negative CD4^−^CD8^−^ Cells by Flow Cytometry

**DOI:** 10.1038/srep05781

**Published:** 2014-07-25

**Authors:** Gang Liu, Zongfang Li, Yang Wei, Yan Lin, Cengceng Yang, Tie Liu

**Affiliations:** 1 The Second Affiliated Hospital of Xi'an Jiaotong University, Xi'an, Shaanxi 710004, China; 2 Clinical Medical Research Center, Affiliated Hospital of Guangdong Medical Collage, Zhanjiang, Guangdong 524001, China; 3 The School of Medicine, Xi'an Jiaotong University, Xi'an, Shaanxi 710061, China; 4 The department of pharmacy, Ningxia medical university, Yinchuan, Ningxia 750004, China

## Abstract

Foxp3 expression is a marker of regulatory T cells (T_reg_), but how early it is expressed in the thymus is still not fully defined. In this study, we examined Foxp3 expression in double-negative (DN) CD4^−^CD8^−^ T cell precursors in the thymus by flow cytometry. By increasing the number of collected cells from the conventional 10^4^ cells up to more than 10^6^ cells during flow cytometry, we found that DN cells exhibited higher Foxp3 expression than double-positive (DP) CD4^+^CD8^+^ and single-positive (SP) CD4^+^ or CD8^+^ (SP) T cells. CD44^+^ expression positively correlated with Foxp3 in thymic DN cells. Furthermore, TCR-β^−^CD25^+^ DN cells exhibited the highest frequency of Foxp3-expressing cells. Almost all Foxp3^+^ cells expressed CD25in DN cells. These results suggest that Foxp3 expression in DN cells can directly be detected by flow cytometry and it was positively corelated with CD25 and CD44 in DN cells.

CD4^+^CD25^+^ regulatory T cells (T_reg_) play a key role in controlling the immune system largely by inhibiting effecter T cell responses both *in vivo* and *in vitro*^1^,^2^. The most reliable marker to identify T_reg_ cells is the transcription factor forkhead box protein P3 (Foxp3)^3^. Double-negative (DN) CD4^−^CD8^−^ cells are the most immature T cell precursors in the thymus. DN cells can be further subdivided by CD44 and CD25 expression into DN1 (CD44^+^CD25^−^), DN2 (CD44^+^CD25^+^), DN3 (CD44^−^CD25^+^), and DN4 (CD44^−^CD25^−^) subpopulations^4^, which then expand and differentiate into mature T cells under cytokine stimulation and interaction with MHC molecules in the thymus^5^,^6^. CD44, an adhesion molecule, is involved in lymphocyte proliferation, maturation, and homing^7^ and its expression positively correlates with TCR-β and Foxp3 expression during T cell development in the thymus^8^.

Recently, the important role of T_reg_ cells in immune regulation has become increasingly recognized, and a large number of studies have focused on T_reg_ cell differentiation, development, and function as well as on its relationship with the Foxp3 transcription factor. While some previous studies show that T_reg_ cells derive from thymic DP cells as a result of increased FoxP3 expression in DP cells^9^, many other reports describe that T_reg_ cells derive from DN cells based on high Foxp3 expression in TCR^−^ DN cells^10^. Since only a small proportion of DP cells are capable of expressing Foxp3, the evidence that T_reg_ cells originate from DP cells is less convincing^11^.

The flow cytometer is widely used analysis tool in biomedical research and clinical diagnostics. It is a powerful tool to study the expression levels of both surface-expressed (such as CD4, CD8, CD44, CD25, TCR-β, etc.) and intracellular (such as Foxp3) molecules on a single-cell basis and the data can be obtained relatively quickly^12^.Using this technique, multiple molecules can be measured at the same time on the same cells, which can help to identify different cell populations within a given sample, and differential expression levels can accurately be measured and compared across various cell populations. In addition to the advantages of using the flow cytometry method in terms of its speed, high precision, and accuracy, the constant hardware updates and improved capability for software-based data analysis make flow cytometry one of the most advanced technologies for cellular quantitative analysis^13^. This technology was later extended in the ImmunoTechnology Section by utilizing quantum dots conjugated to monoclonal antibodies, upgraded instrumentation and allowed measurements up to 18 different colors can be detected by flow cytometers, making it the only immunological technique allowing simultaneous determination of T cell function and phenotype. In addition, cell proliferation and viability can be also measured^14^.

In this study, we used flow cytometry to examine Foxp3, CD25, and CD44 expression in DN thymocytes. By increasing the normal sample collection number of 10^4^ thymocytes up to more than 10^6^ thymocytes, we found that CD44 expression positively correlated with TCR-β and Foxp3 expression in DN cells. Furthermore, CD25 expression positively correlated with Foxp3 and negatively correlated with TCR-β in DN cells, thus, our data describe the relationship among Foxp3, CD25, and CD44 cell surface markers on DN thymocytes. The study reported here had two aims. First, this study evaluated the levels of Foxp3 expression in DN cells by the improved detection mode of Flow cytometry. Second, the efficiency and success of the improved detection mode was compared to traditional mode, it may help us to better understand T_reg_ cell development in the thymus.

## Methods

### Mice

Female C57BL/6 mice (Taconic Farms, Germantown, NY) were maintained under specific pathogen-free conditions and used for experimentation at 4–6 weeks (young) and 6–10 months (adult) of age according to protocols approved by the Institutional Animal Care and Use Committee at Xi'an Jiaotong University.

### Flow cytometric analysis and cell sorting

Thymocytes were obtained from naïve mice and suspended in phosphate-buffered saline (PBS) plus 1% fetal calf serum (FCS). To avoid non-specific binding to mouse Fcγ receptors, cells were blocked with mouse CD16/CD32 mAb (0.25 μg/100 μL) (BD Biosciences, Franklin Lakes, NJ) for 15 min at room temperature. After washing, cells were stained with PE-Cy7–anti-CD4 (clone RM 4–5), FITC–anti-CD8α (clone 53-6.7), PE–anti-CD44 (clone 1M7), APC-AlexaFluor 755–anti-CD25 (clone PC-61.5), or PerCP–TCRβ (clone H57-597) antibodies for 60 min at 4°C. In some cases, the surface-stained cells were fixed/permeabilized with a Cytofix/Cytoperm kit (BD Biosciences) and then stained with a PE-Cy5–anti-Foxp3 (clone FJK-16s, eBioscience) antibody for 45 min at 4°C. The corresponding isotype controls (rat IgG1, IgG2a, and IgG2b) were purchased from eBioscience (San Diego, CA) and BD Biosciences. Cells were analyzed using a FACSCalibur flow cytometer or FACSAria cell sorter (BD Biosciences) and the data were analyzed using BD FACSDiva software (BD Biosciences).

To conform whether DN cells express higher levels Foxp3, the cells were also stained with FITC–anti-CD4, PerCP5.5–anti-CD8 and PE-anti-Foxp3 antibodies (eBiosciences).

### RT-PCR and real-time RT-PCR

Total RNA was extracted from ~5 × 10^5^ sort-purified thymocytes of C57BL/6 mice, using TRIzol (Invitrogen). Chloroform (0.2 ml; Sigma- Aldrich) was added for every 1 ml of TRIzol used. Extracted RNA was shaken vigorously for 15 sec and incubated at room temperature for 2 min. After transferring the aqueous phase to a clean tube, isopropanol (Sigma-Aldrich) was added, followed by incubation at room temperature for 5 min. The RNA pellet was washed with 1 ml of 75% ethanol. After air-drying the RNA pellet for 10 min, it was dissolved in 20 μl of water and incubated at 55°C for 10 min. The OD260 nm/280 nm ratio was among 1.9 to 2.0. RNA samples were further assessed by electrophoresis on 1.5% agarose gels, and then visualized under UV light after ethidium bromide staining. RNA preparations were treated with DNase I (according to the standard protocol) to remove genomic DNA. cDNA was synthesized by incubating 20 μl of mRNA in a sprint C1000 terminal cycler (Bio-rad). Negative controls contained all the elements of the reaction mixture except for template DNA. For quantification, relative mRNA expression of specific genes was obtained by the 2^−ΔΔCt^ method, using β-actin for normalization. The following gene-specific primers (5′ → 3′) were used: β-actin (forward; GA*A ATC GTG CGT GAC ATC AAA G*, and reverse; TG*T AGT TTC ATG GAT GCC ACA G*); Foxp3 (forward; GG*C CCT TCT CCA GGA CAG A*, and reverse; GC*T GAT CAT GGC TGG GTT GT*). Diluted cDNA (10 μl) was mixed with 2 μl of primer and 10 μl IQ SYBR Green SuperMix, and was then assayed in triplicate on a CFX-96 real-time system (Bio-Rad) under the following conditions: denaturation at 95°C for 3 min, 40 cycles of 95°C for 15 s, and 60°C for 1 min followed by 30 sec of extension at 72°C. Each sample was analyzed in triplicate.

### Statistical analysis

Mean and SD values were calculated with Microsoft Excel. At least three independent experiments were performed, the Tukey-Kramer post-test was used to compare 3 or more means or a two-tailed unpaired Student *t* test to compare 2 groups, Values of *P* ≤ 0.05 were considered significant. Statistically significant values are denoted in the figures as follows: *P < 0.05; **P < 0.01; ***P < 0.001.

## Results

### Foxp3 expression is detected in thymic double-negative CD4^−^CD8^−^ cells by flow cytometry

**Figure 1:**
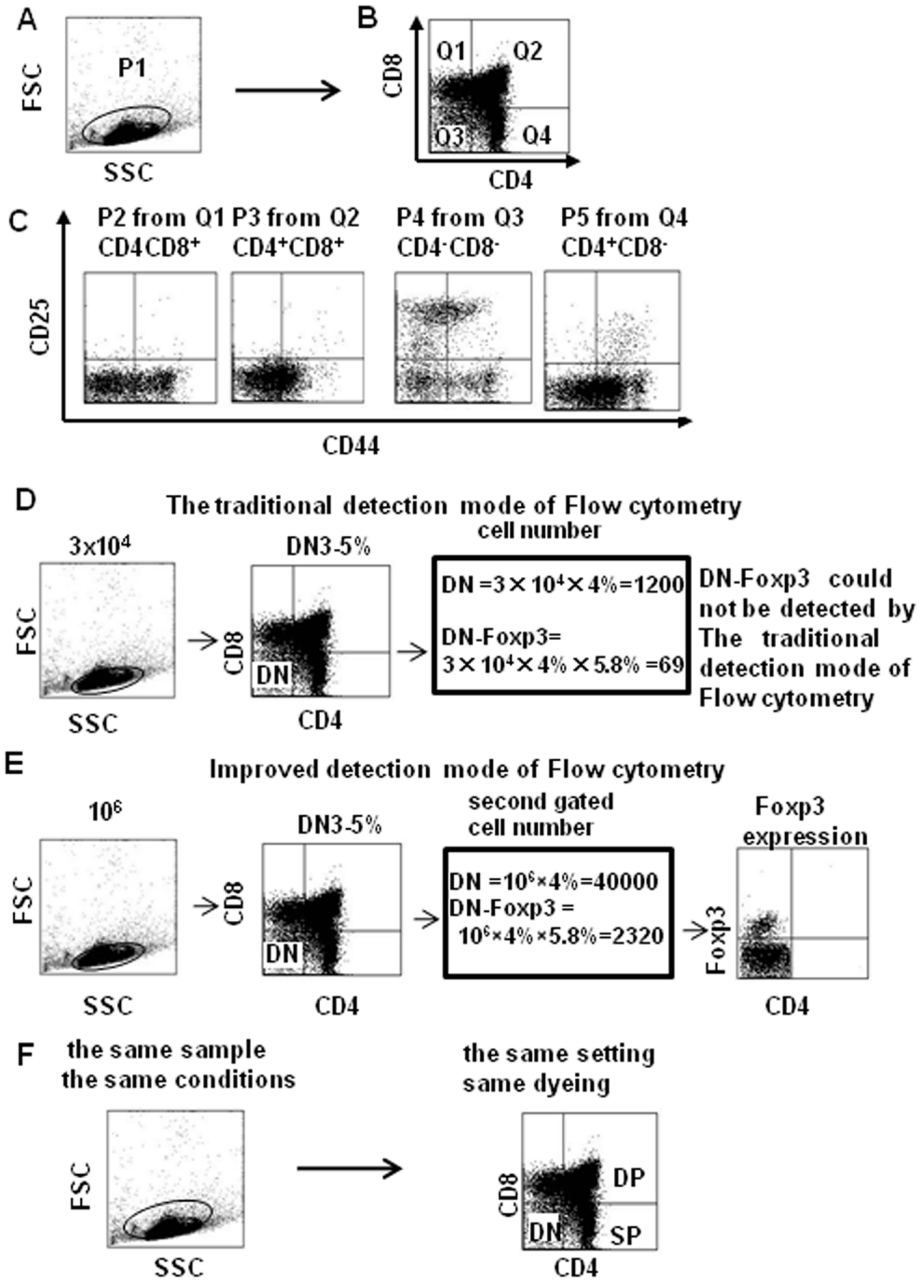
Detection of Foxp3 expression in DN cells by flow cytometry. Thymocytes were obtained from naïve C57BL/6 mice and stained with specific antibodies against CD4 (PE-Cy7), CD8 (FITC), CD44 (PE), CD25 (APC-AlexaFluor 755), TCR-β ( PerCp ) and Foxp3 (PE-Cy5). (A) Cell-gating strategy and staining patterns. Cells in P1 were gated as live thymocytes. (B) Based on CD4 and CD8 expression, four subpopulations (P2 to P5) were defined by the four quadrants (Q1 to Q4). (C) The DN subpopulation within the thymocytes (Q3) was evaluated for CD25 and CD44 expression. (D) The traditional detection mode of Flow cytometry. (E) Improved flow cytometry method. (F) High repeatability in the new mode.

DN cells only comprise 3–5% of the total thymocytes within the thymus^15^,^16^, and even less of these DN cells express Foxp3; therefore, accurately detecting Foxp3 expression in DN cells by flow cytometry is difficult, and no studies have yet reported direct detection of Foxp3 expression in DN cells by this method. In order to increase the sensitivity of detecting Foxp3 in this small population of cells, we first increased the cell collection number for each sample from 10^4^ up to more than 10^6^ and examined the developmental profile of thymocytes in mice using multi-color flow cytometry. Thymocytes from naïve mice were stained with the following combination of antibodies: PE-Cy7–anti-CD4, FITC-anti–CD8, APC-Cy7–anti-CD25, PE–anti-CD44, PE-Cy5–anti-Foxp3, and PerCp–anti-TCR-β. As illustrated in Figure 1, we first gated live thymocytes by size (FSC) and granularity (SSC) in the P1 gate Figure 1A, followed by dividing the live cells into four subpopulations based on CD4 and CD8 expression, which are identified as quadrants Q1–Q4 Figure 1B. The P2 population represents Q1 (CD4^−^CD8^+^, or CD8^+^ SP), P3 represents Q2 (CD4^+^CD8^+^, or DP), P4 represents Q3 (CD4^−^CD8^−^, or DN), and P5 represents Q4 (CD4^+^CD8^−^, or CD4^+^ SP). Since the cells had also been stained with antibodies against CD44 and CD25 Figure 1C.

The expression of many genes, such as Foxp3 are low in CD4^−^CD8^−^ (double negative, DN) thymocytes; The cell collection numbers in the traditional detection mode of Flow cytometry are 3 × 10^4^, the cell collection numbers of DN cells will be 1200 (3 × 10^4^ × 4%), DN-Foxp3 cells will be 69 (3 × 10^4^ × 4% × 5.8%), therefore, DN-Foxp3 could not be detected by Flow cytometry, because the cell collection numbers of DN cells are too small (Figure 1D). We increased the cell collection number for each sample from 10^4^ up to more than 10^6^, the cell collection numbers of DN cells will be 40000(10^6^ × 4%), the second gated DN-Foxp3 cell number will be 2320 (10^6^ × 4% × 5.8%) (Figure 1E). The three populations of thymus from the same sample which is the same conditions, the same setting and same dyeing (Figure 1F). Therefore, using this improved flow cytometry method, lower expression genes in DN cells could be detected high repeatability to reveal previously uncharacterized data on subsets of DN cells. We greatly improved the accuracy of Foxp3 detection in DN cells.

### Foxp3 expression correlates with CD25 expression in DN cells

**Figure 2:**
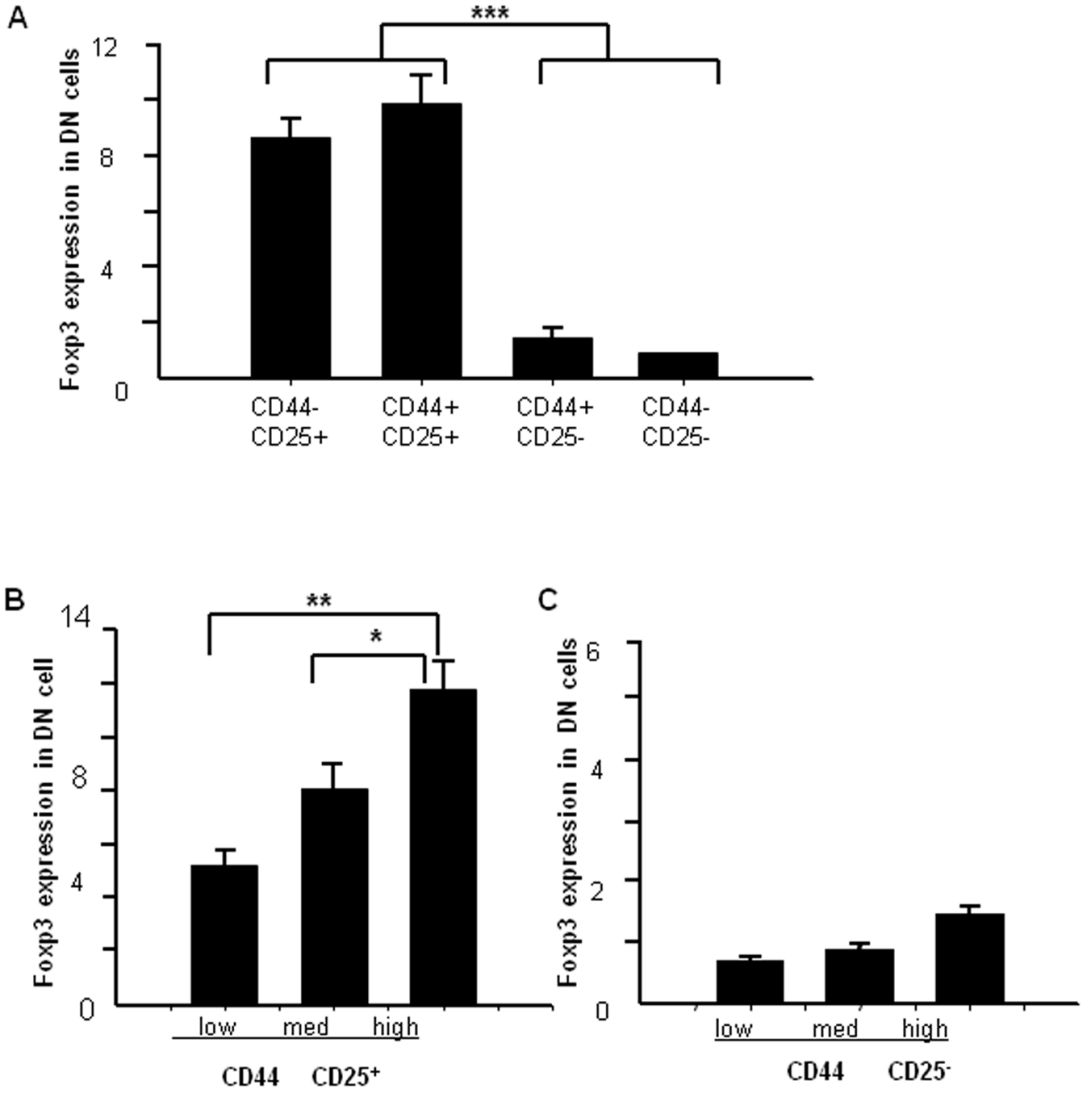
Foxp3 expression in the various thymic DN subpopulations. (A) Cells were stained with CD4 (PE-Cy7), CD8 (FITC), CD44 (PE), CD25 (APC-AlexaFluor 755), and Foxp3 (PE-Cy5) expression in CD44^−^CD25^−^, CD44^−^CD25^+^, CD44^+^CD25^−^, and CD44^+^CD25^+^ DN cells was analyzed. (B) Foxp3 in CD44^+^CD25^+^ DN cells according to varying CD44 expression levels. (C) Foxp3 in CD44^+^CD25^−^ cells according to varying CD44 expression levels. The data show the percentage of total and live thymocytes (population P1) in each cell subset, and are presented as the mean ± SD from three independent experiments. (*P < 0.05, **P < 0.01, ***P < 0.001).

We further analyzed DN cells that were gated according to their CD25 and CD44 expression by flow cytometry. CD25^+^ cells had a higher frequency of Foxp3-expressing cells (CD25^+^CD44^−^ DN, 8.2%; CD25^+^CD44^+^ DN, 10.3%) than CD25^−^ cells (CD25^−^CD44^−^ DN, 0.2%; CD25^−^CD44^+^ DN, 0.7%) (Figure 2A). Further analyzing the CD25^+^CD44^+^ DN subpopulation by the differential CD44 expression, CD25^+^CD44^high^ cell had the highest frequency of Foxp3-expressing cells (12.7%), followed by CD25^+^CD44^med^ (8.1%), and CD25^+^CD44^low^ (4.6%) (Figure 2B). In contrast, cells expressing differing levels of CD44 in the CD25^−^ subpopulation exhibited very low Foxp3 expression (Figure 2C). These results suggest that Foxp3 expression in CD25^+^, but not CD25^−^, DN cells that also express CD44 positively correlate with Foxp3 expression.

### Foxp3 correlates with CD25 expression in other thymocyte subpopulations

**Figure 3:**
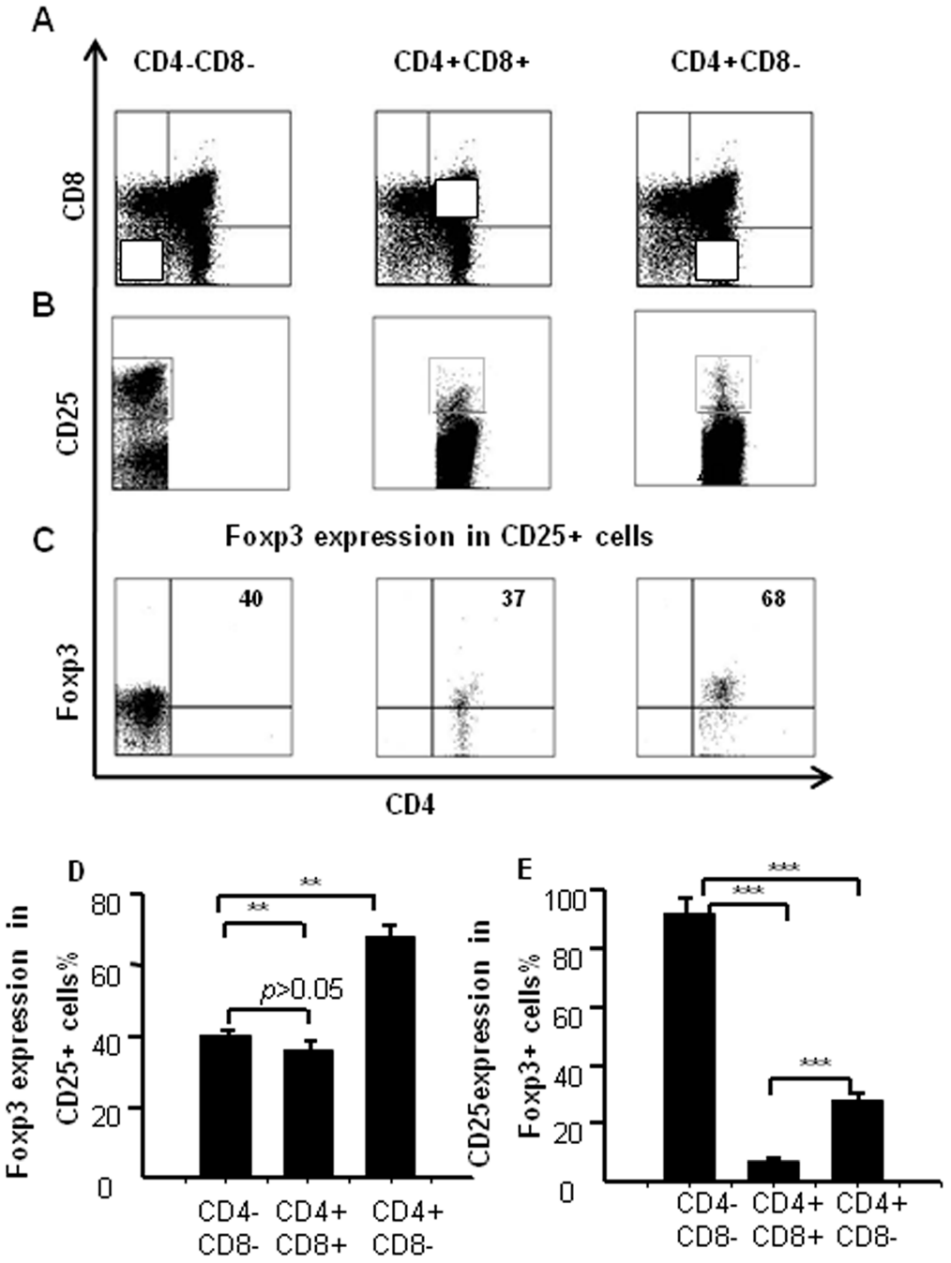
Foxp3 correlates with CD25 expression in other thymocyte subpopulations. (A) Cells were stained as in Figure 2, and CD25 expression was analyzed in the different subpopulations of mouse thymocytes. (B) Foxp3 expression was evaluated within the CD25^+^ gate from thymic DN, DP, and SP cells. (C) Expression of Foxp3 in CD25^+^ cells; (D) data were pooled from three independent experiments and are shown in the plot as average mean ± SD. (E) Expression of CD25 in Foxp3^+^ cells. The data shown represent the percentages of total and live thymocytes in each cell subset, and are presented as the mean ± SD from three independent experiments. (**P < 0.01, ***P < 0.001).

To confirm whether Foxp3 expression correlates with CD25 expression in the other thymocyte subpopulations, thymocytes from naïve mice were stained with the following combination of antibodies: PE-Cy7–anti-CD4, FITC-anti–CD8, APC-Cy7–anti-CD25, PE-Cy5–anti-Foxp3, As illustrated in Figure 3A, we gated on CD25^+^ cells from the DN, DP, and SP subpopulations and analyzed Foxp3 expression (Figure 3B). CD4^+^CD25^+^ SP cells expressed higher Foxp3 levels (68%) than CD25^+^ DN cells (40%) and CD25^+^ DP cells (40%) (Figure 3C). Figure 3D shows the pooled data from three independent experiments. Gating on Foxp3^+^ cells, we analyzed CD25 expression from each subpopulation and found that almost 100% of Foxp3^+^ cells expressed CD25 in DN cells (Figure 3E). These results suggest that Foxp3 expression positively correlates with CD25 and cell maturation states.

### Foxp3 expression during different stages of thymocyte development in adult and young mice

**Figure 4:**
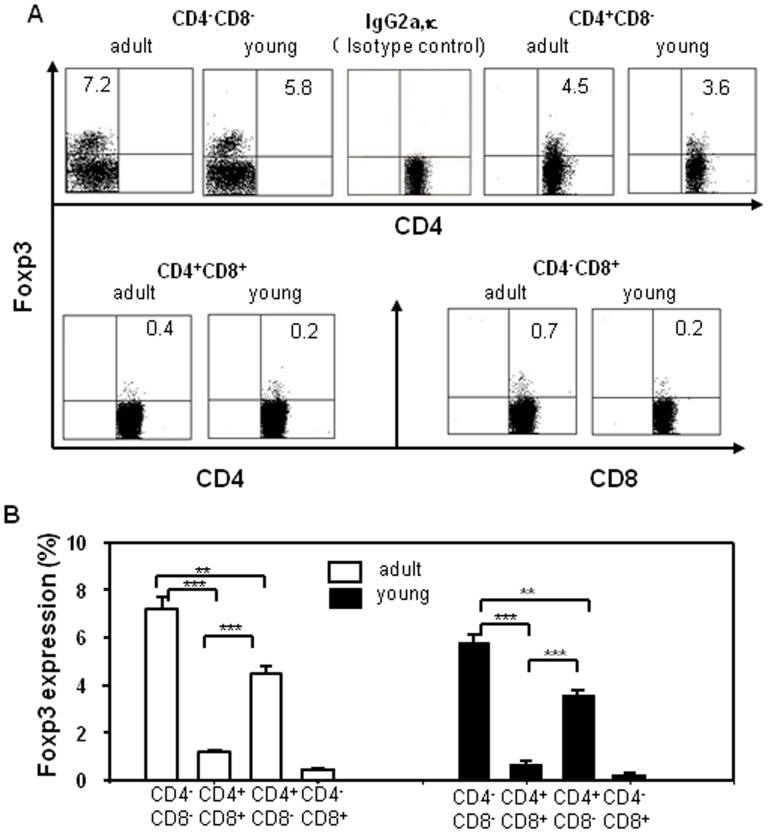
Foxp3 expression in the thymocytes of adult and young mice. Thymocytes were collected from naïve mice, stained with antibodies against CD4 (PE-Cy7), CD8 (FITC), CD44 (PE), CD25 (APC-AlexaFluor 755), Foxp3 (PE-Cy5) and Isotype IgG2a, and analyzed by flow cytometry. (A) Foxp3 expression in the thymocytes of adult and young mice. (B) The data shown were pooled from three independent experiments. The data show the percentage of total cells, and are presented as the mean ± S.D. from three independent experiments. (**P < 0.01, ***P < 0.001).

Thymocytes were harvested from young and adult mice, stained with CD4 (PE-Cy7), CD8 (FITC), CD44 (PE), CD25 (APC-AlexaFluor 755), Foxp3 (PE-Cy5) and Isotype IgG2a as negative control (Foxp3) and analyzed by flow cytometry. The frequency of Foxp3-expressing cells was higher in adult mice than in young mice. Among the thymocyte subpopulations, DN cells had a much higher frequency of Foxp3-expressing cells in both adult and young mice (adult, 7.2%; young, 5.8%), followed by CD4^+^ SP cells (adult, 4.5%; young, 3.6%), CD8^+^ SP cells (adult, 0.7%; young, 0.2%), and DP cells (adult, 0.4%; young, 0.2%) (Figures 4A and 4B). Thus, our results reveal that although Foxp3 is a T_reg_ cell marker, it is also a marker that dynamically changes in thymocytes. DP cells contained the lowest frequency of Foxp3-expressing cells in thymocytes. These results further suggest that Foxp3 expression correlates with cell maturation states.

**Figure 5:**
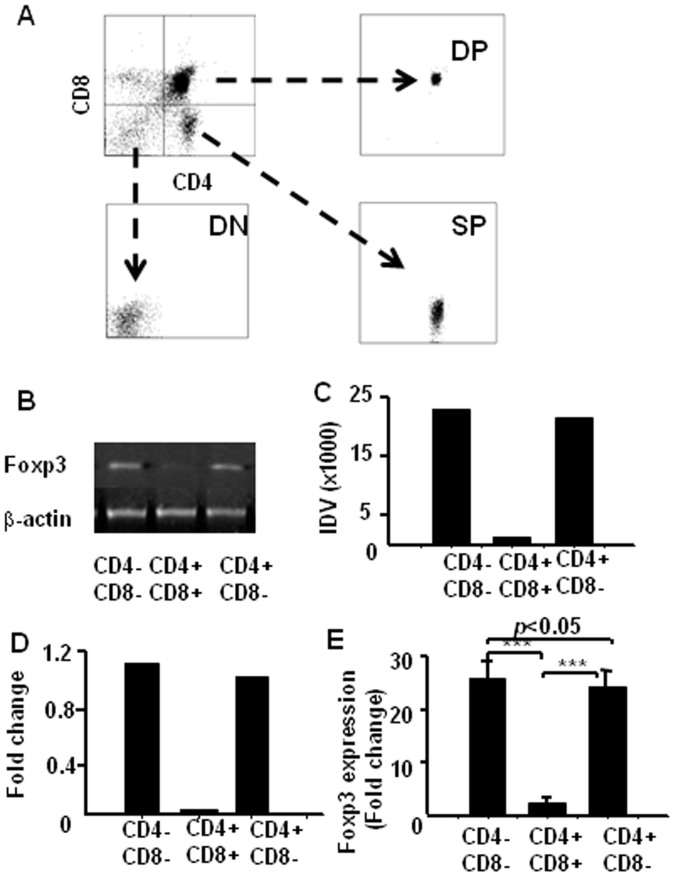
Foxp3 expressions in the thymocytes by RT-PCR and Real-time-RT-PCR. CD4^−^CD8^−^, CD4^+^CD8^+^ and CD4SP cells were sorted from the thymocytes of C57BL/6 mice (A), total RNA was extracted for RT-PCR and Real-time-RT-PCR analysis of Foxp3 expression. (B) Foxp3 expression in the thymocytes by RT-PCR. (C) The integrated density values (IDV) for the Foxp3 transcripts were quantitated and normalized to those of β-actin. Shown are representative results from one of three independent experiments. (D) The data were pooled from three independent experiments and shown in the plot. (E) Total RNA was isolated for measuring Foxp3mRNA by real-time RT-PCR. Data are presented as fold-induction relative to the levels of β-actin. Shown are representative results one of three independent experiments. (*** *p* < 0.001).

To conform whether DN cells express higher levels Foxp3, we sorted DN, DP and CD4^+^SP cells from the thymocytes by FACS (Figure 5A). We examined *via* RT-PCR the levels of Foxp3 mRNA, the levels of Foxp3 were higher in DN and SP cells than DP cells as determined by densitometry scanning of the gels (Figure 5B) and the data were pooled from three independent experiments and shown in a plot (Figure 5C and 5D). To further confirm these findings, we sorted DN, DP and CD4^+^SP cells from the thymocytes and measured the levels of Foxp3 mRNA by real-time RT-PCR. As shown in (Figure 5E), DN and CD4^+^SP cells expressed higher levels of Foxp3 than DP cells (more than 20 times). These results further suggest that Foxp3 expression was much higher in DN cells but not in DP cells.

## Discussion

In this study, we directly detected the expression of Foxp3 in thymic DN cells by multi-color flow cytometry and observed that DN and CD4^+^ SP cells contained a higher frequency of Foxp3-expressing cells than DP cells. We also found that CD44 expression positively correlated with Foxp3 that CD25^+^ DN cells expressed higher Foxp3 levels, and that CD25^−^ DN cells do not expressed Foxp3.

We used multi-color flow cytometry to analyze the thymic DN cell population. We determined surface marker expression on thymocytes and found that Foxp3 expression changed in different stages of T cell development. One previous study did not find expression in DN cells by the RT-PCR method^17^; other used MACS to isolate DN cells from thymocytes and remove any contaminating NK, B, and macrophage, but this group also did not observe any Foxp3 expression in DN cells^18^. By increasing the cell collection from the normally used 10^4^ cells up to more than 10^6^ cells during the acquisition phase of flow cytometry, we were able to identify a Foxp3-expressing population within DN cells and were further able to analyze its expression in each of the DN cell subpopulations (Figure 1). CD25^+^ DN cells expressed higher Foxp3 levels; whereas CD25^−^ DN cells expressed much lower Foxp3 levels (Figure 1D). Furthermore, Foxp3 also positively correlated with CD44 (Figure 2). The frequency of Foxp3-expressing cells was much higher in DN cells than in DP or SP cells. Among the CD25^+^ cells specifically, CD4^+^ SP cells had the highest frequency of Foxp3-expressing cells compared to the DN and DP cells, which were similar to each other (Figure 3C). These results tell us that Foxp3 expression positively correlates with CD25 expression and the state of cell maturation. Almost 100% of Foxp3^+^ cells were CD25^+^ in DN cells, while only 4.3% of Foxp3^+^ cells were CD25^+^ in DP cells; meanwhile, 34% of Foxp3^+^ cells were CD25^+^ cells in SP cells (Figure 3E). Our experiment testing Foxp3 expression in thymocytes from young and adult mice also suggested that Foxp3 expression correlated with cell maturation status (Figure 4). To conform whether DN cells express higher levels Foxp3, we sorted DN, DP and CD4^+^SP cells from the thymocytes by FACS. We examined the levels of Foxp3 *via* RT-PCR and real time RT-PCR (Figure 5). Our data also showed that CD25 expression negatively correlated with TCR-β expression. This raises the question of whether some function of CD25 may interrupt TCR signaling transformation through Foxp3 expression. Perhaps TCR signaling controls Foxp3 expression^19^, while Foxp3 or CD25 expression can also interfere with TCR signaling? Also, how do CD25^+^ DN cells express Foxp3 to seed development of T_reg_ cells in thymus? This is a very interesting question. T_reg_ cells may originate from DN cells, where they do not express TCR; once they move into the CD4^+^ SP stage, they express TCR, but this TCR is non-functional. Consistent with this, neither anti-CD3 nor anti-TCR can induce T_reg_ cell proliferation^20^, but anti-CD25 mAb treatment can not only block T cell activation but also prevent activation-induced cell death^21^. The precise functional role of CD25 in the TCR signaling pathway and its relationship with Foxp3 expression is currently unclear and requires further study. In order to enhance the link between the Foxp3-expressing DN cells, perhaps consider also including some functional tests. We could sort these Foxp3-expressing DN cells and evaluate their capability in vitro or in vivo to give rise to functional T_reg_ cells that can suppress the proliferation of activated CD8+ cells.

In this study, we determined a reliable method for analyzing the relationship among Foxp3, CD25, and CD44 expression in DN cells by increasing the cell collection number from 10^4^ up to more than 10^6^ thymocytes, the approach is standards method of flow cytometry, we apply the new protocol to detect T cell base on BD software which is modern FACS data analysis software has introduced easily accessible compensation utilities that simply make fluorescence compensation the first part of the analysis procedure with any primary FACS data set. Since primary data can be collected with any FACS instrument just by avoiding the compensation step^22^. Today's FACS technology readily supports the collection of primary data and frees us to do better and more accurate analyses. In facts, it can directly clear detect to the expression of Foxp3 in DN cells. In order to improve the detection efficiency, we should pay attention to: (i) in our experiment, the data of capacity in computer very large, a specimen is equivalent to 30–100 times normal, it was very slowly when we did compensation correction, so we had prepare one sample to collect 10^4^ thymocyte s, specially for compensation correction; (ii) Multiple antibody can not be together staining, especially the lower expression of gene should be used to separate; (iii) This study used the off-line compensation technology, it needs very careful for repeated verification accuracy of compensation.

In summary, characterization of DN cells is technically difficult, because the very small number of DN in mice thymus. In the present study, we have developed a reliable and efficient multiple -color flow cytometry method. We reveal that DN cells have a higher frequency of Foxp3-expressing cells than DP or CD4/CD8 (SP) cells; CD44^+^ DN cells express Foxp3; CD25^+^ DN cells express Foxp3; and CD25^−^ DN cells do not expressed Foxp3. Taken together, our results suggest Foxp3 expression in DN cells can directly be detected by flow cytometry and Foxp3 expression was positively corelated with CD25 and CD44 in DN cells.
